# Cell-based immunotherapy approaches for multiple myeloma

**DOI:** 10.1038/s41416-018-0346-9

**Published:** 2018-12-06

**Authors:** Katharina Kriegsmann, Mark Kriegsmann, Martin Cremer, Michael Schmitt, Peter Dreger, Hartmut Goldschmidt, Carsten Müller-Tidow, Michael Hundemer

**Affiliations:** 1 Department of Hematology, Oncology and Rheumatology, Heidelberg University, Heidelberg, Germany; 2 Institute of Pathology, University Hospital Heidelberg, Heidelberg, Germany; 3 National Center of Tumor Diseases (NCT), University Hospital Heidelberg, Heidelberg, Germany

**Keywords:** Myeloma, Molecular medicine, Myeloma

## Abstract

Despite the arrival of novel therapies, multiple myeloma (MM) remains incurable and new treatment options are needed. Chimeric antigen receptor (CAR) T cells are genetically modified T cells that express a CAR directed against specific tumour antigens. CAR T cells are able to kill target tumour cells and may result in long-lasting immune responses in vivo. The rapid development of CAR technologies has led to clinical trials in haematological cancers including MM, and CAR T cells might evolve into a standard treatment in the next few years. Only small patient cohorts with relapsed or refractory disease have so far been investigated, but promising preliminary results with high response rates have been  obtained in phase I clinical trials with B cell maturation antigen (BCMA), CD19, CD38 and κ-light-chain CAR T cells. Additional preclinical studies on CD38 and SLAMF7-CAR T cells in MM treatment yielded preclinical results that merit further investigation. Beyond the T cell approach, recent studies have focussed on CAR natural killer (NK) cells in order to increase the reactivity of these effector cells. Finally, to investigate the targeting of intracellular antigens, cellular therapies based on engineered T cell receptors (TCRs) are in development. In this review, we discuss results from preclinical and early-phase clinical trials testing the feasibility and safety of CAR T cell administration in MM, as well as early studies into approaches that utilise CAR NK cell and genetically modified TCRs.

## Introduction

With an incidence of approximately 5 cases per 100,000 persons per year, multiple myeloma (MM) accounts for around 10% of all haematological malignancies.^1^ Introduction of novel agents, such as thalidomide, bortezomib, and lenalidomide, was one of the major advances in frontline MM treatment during the past decade.^2^ For induction treatment, transplant-eligible patients usually receive several cycles of three-drug regimens (dexamethasone, bortezomib, and doxorubicin or cyclophosphamide or an immunomodulatory drug (IMiD)). Upon successful peripheral blood stem cell collection, high-dose melphalan therapy is given and autologous stem cell transplantation (ASCT) is performed, followed by lenalidomide maintenance therapy.^3^ Treatment choice at relapse is guided by clinical variables including performance status and age, as well as previous therapeutic lines and time to relapse. At first relapse after IMiD-based induction, doublet therapy with Kd (carfilzomib, low-dose dexamethasone) or Vd (bortezomib, low-dose dexamethasone), or triplet therapy based on bortezomib are approved. At first relapse after bortezomib-based induction, Rd (lenalidomide, low-dose dexamethasone) or Rd-backbone triplets are available.^3^ Despite novel treatment options, MM remains incurable and  new treatment options are needed. According to the revised international staging system (R-ISS) for prognosis, the 5-year OS is 82, 62 and 40% for R-ISS stage I, II and III, respectively.^4^

Several tumour-associated antigens and immune escape mechanisms have been identified in MM, meaning immunotherapy is a promising treatment option, particularly in cases of relapsed/refractory disease. Chimeric antigen receptor (CAR) T cell immunotherapy has evolved as a potential anti-cancer therapy^5^ that has shown promising results in early clinical trials and, following the high response rates achieved in patients with pretreated and chemotherapy-resistant chronic and acute leukaemia and lymphoma, the FDA approved the application of CAR T cells in refractory CD19^+^ acute lymphoblastic leukaemia and diffuse large B cell lymphoma.^6–10^ Currently, CAR T cell therapy is being investigated in additional haematological neoplasia such as relapsed/refractory MM, where it has shown high remission rates and prolongation of progression-free survival.^6,11–14^

## CAR T cells in the treatment of MM

CAR T cells are genetically modified T cells that express a chimeric antigen receptor (CAR). As CAR T cells are generated from autologous T cells collected from patients by leukapheresis, they represent an individualised therapy concept.^15^ Similar to monoclonal antibodies (mAbs), CARs are directed against specific cell-surface antigens, which should ideally only be expressed on target (tumour) cells and not on healthy tissues, to limit toxicity.^16^ Unlike mAbs, CAR T cells are not only able to kill target cells but might also induce a long-lasting immune response against the target antigen due to their  persistence in vivo, aided by prior lymphodepletion by chemotherapy and radiotherapy to create a CAR T cell niche.^5,17–19^

### Anti-tumour activity of CAR T cells in early clinical trials

The use of CAR T cells in the treatment of MM is currently limited to a few antigens and early-phase clinical trials. The B cell maturation antigen (BCMA) is particularly suitable as a CAR T cell target in MM, because it is specifically expressed on cells of the B lineage, including plasma cells and myeloma cells.^20^ BCMA is a member of the tumour necrosis factor receptor superfamily and is bound by ligands such as B cell-activating factor of the tumour necrosis factor family (BAFF) and a proliferation-inducing ligand (APRIL), which, as their names imply, induce B cell activation and proliferation.^21^ CAR T cells that target BCMA are currently being investigated in phase I clinical trials, and the preliminary results achieved in relapsed/refractory MM with regard to safety and efficacy are promising (Table 1).

**Table 1:** CAR T cells in multiple myeloma early-phase clinical trials

Antigen/ Reference	Trial design	CAR construct/ vector	CAR T cell dose	Conditioning/ lymphodepletion	Patients reported	Safety/side effects	Anti-tumour activity
*BCMA*							
Ali et al.,^22^ Brudno et al.^23^ National Cancer Institute, National Institutes of Health, Bethesda	Phase I dose-escalation study	Costimulation: CD28 Vector: γ retroviral	0.3–9 × 10^6^ cells/kg body weight	Cyclophosphamide (3 × 300 mg/m^2^) and fludarabine (3 × 30 mg/m^2^)	r/r MM, *n* = 27	CRS and prolonged cytopenias in patients treated on the 9 × 10^6^ cells/kg dose level	Anti-tumour activity of BCMA-CAR T cells in poor-prognosis MM demonstrated
Cohen et al. ^24, 25^ University of Pennsylvania, Philadelphia	Phase I dose-escalation study	Costimulation: 4-1BB Vector: lentiviral	DL 1: 1–5 × 10^8^ cells DL 2: 1–5 × 10^7^ cells DL 3: 1–5 × 10^8^ cells (absolute number)	DL 1: none DL 2/3: cyclophosphamide 1.5 g/m^2^	r/r MM, *n* = 21	CRS (*n* = 17), severe reversible neurotoxicity (*n* = 3)	Promising in vivo CAR T cell expansion and clinical activity, even without lymphodepletion Depth of response correlates with degree of BCMA-CAR T cell expansion and CRS
Berdeja et al.^26, 27^ Multicentre	Multicentre phase I dose-escalation study	Costimulation: 4-1BB Vector: lentiviral	50–1200 × 10^6^ cells (absolute number)	Cyclophosphamide (3 × 300 mg/m^2^) and fludarabine (3 × 30 mg/m^2^)	r/r MM, *n* = 21	CRS (*n* = 17)	Promising efficacy (100% ORR) at dose levels above 50 × 10^6^ cells
Smith et al.^28^ Memorial Sloan-Kettering Cancer Center, New York	Phase I dose-escalation study	Costimulation: 4-1BB Vector: retroviral	DL 1: mean 72 × 10^6^ cells DL 2: mean 137 × 10^6^ cells (absolute number)	DL 1: cyclophosphamide (1 × 3 g/m^2^) DL 2: cyclophosphamide/ fludarabine (3 × 300/30 mg/m^2^)	r/r MM, *n* = 6	CRS grade 1–2 (*n* = 3)	Promising anti-tumour activity in highly pretreated patients
Mi et al.,^31^ Fan et al.^30^ Shanghai Institute of Hematology, Shanghai Jiao Tong University, Shanghai	Phase I	Antigen recognition: bi-epitope Costimulation: not published vector: not published (LCAR-B38M)	Median 4.7 × 10^6^ cells/kg BW infused over 3 days	Cyclophosphamide (3 × 250 mg/m^2^) and fludarabine (3 × 25 mg/m^2^)	r/r MM, *n* = 19	CRS (*n* = 14)	Objective response achieved in all patients, CR/nCR in 19 patients
*CD19*							
Garfall et al.^33, 34^ University of Pennsylvania, Philadelphia	Phase I	Costimulation: 4-1BB Vector: lentiviral (CTL019)	1–5 × 10^7^ cells (absolute number)	Melphalan (140–200 mg/m^2^) and ASCT	r/r MM, *n* = 10	Most toxicity attributable to ASCT, no severe CRS	CTL019 may prolong response of standard MM therapies
*CD138*							
Guo et al.^38^ General Hospital of PLA, Beijing	Phase I	Costimulation: 4-1BB Vector: lentiviral	0.756 × 10^7^ cells/kg BW	CP or PCD or VAD	r/r MM, *n* = 5	No intolerable toxicities, grade 3 fever upon CAR T cell infusion	Feasibility demonstrated, stable disease in four patients longer than 3 months
*κ-light-chain*							
Ramos et al.^39^ Baylor College of Medicine, Houston	Phase I	Costimulation: CD28 Vector: retroviral	0.2–2 × 10^8^ cells/m^2^ BS	Cyclophosphamide (12.5 mg/kg) in patients without lymphopenia	r/r MM, *n* = 7	No toxicities attributable to CAR T cells	Stable disease 4 patients lasting 2–17 months
*NKG2D ligands*							
Nikiforow et al.^43^ Dana-Farber Cancer Institute, Boston	Phase I dose-escalation study	Receptor design: NKG2D, DAP10 signal transmission subunit, CD3ζ Signalling domain vector: retroviral (CM-CS1)	1 × 10^6^–3 × 10^7^ cells (absolute number)	None	r/r MM, *n* = 5	Safety demonstrated, no dose-limiting toxicity	Feasibility demonstrated

*ASCT* autologous stem cell transplantation, *BCMA* B cell maturation antigen, *BW* body weight, *BS* body surface, *CAR* chimeric antigen receptor, *CRS* cytokine-release syndrome, *DL* dose level, *MM* multiple myeloma, *(n)CR* (near) complete response, *ORR* overall response rate, *r/r* relapsed/refractory

Literature research was mainly based on the ASH annual meeting abstracts considering the search terms “CAR/chimeric antigen receptor and multiple myeloma” from all years (number of screened abstracts >300). The table makes no claim to be comprehensive

Ali et al.^22^ and Brudno et al.^23^ published the first results of a phase I dose-escalation trial of BCMA-CAR T cell treatment (0.3–9 × 10^6^ CAR T cells/kg body weight) in 27 patients with relapsed/refractory MM, in which the anti-tumour activity of BCMA-targeted CAR T cells in poor-prognosis MM was demonstrated, using a cyclophosphamide/fludarabine conditioning regimen. Cytokine-release syndrome (CRS) and prolonged cytopenia occurred in patients treated with the 9 × 10^6^ CAR T cells/kg dose.^22,23^ Cohen et al.^24^ carried out a phase I dose-escalation study using a fully human BCMA-specific CAR with CD3ζ and 4-1BB signalling domains, the results of which showed promising in vivo CAR T cell expansion and clinical activity in 21 highly pretreated MM patients, even without lymphodepletion. CRS, characterised by increased levels of circulating cytokines such as interleukin-6 (IL-6), was reported in 17 patients (six of whom showed CRS grade 3–4) and severe reversible neurotoxicity was reported in three patients. Interestingly, the depth of response correlated with the degree of BCMA-CAR T cell expansion and CRS.^25^ In a separate study, Berdeja et al.^26,27^ treated 21 relapsed/refractory MM patients in a multicentre phase I dose-escalation trial with a second-generation BCMA-targeted CAR T cell construct upon lymphodepletion with fludarabine and cyclophosphamide, and reported manageable CRS, no dose-limiting toxicities, and promising anti-MM efficacy at dose levels above 50 × 10^6^ CAR T cells, achieving an overall response rate (ORR) of 100%. Similarly, Smith et al.^28,29^ reported promising results in a small cohort of six patients with relapsed/refractory MM treated with BCMA-CAR T cells. Using a technique known as bi-epitope targeting, Fan et al.^30^ and Mi et al.^31^ reported on the clinical application of CAR T cells engineered to target two distinct regions of BCMA in a cohort of 19 relapsed/refractory MM patients. CRS was reported in 14 patients and was manageable. Of particular interest, a 100% ORR was achieved and 18 of the patients (95%) reached complete remission or near-complete remission. No relapses were observed at a median follow-up of 6 months.^30,31^

Although usually expressed on B cells, the B cell co-receptor CD19 can also be found on a small proportion of myeloma cells that might represent MM cancer stem cells.^15^ In a 2014 phase I clinical trial of 10 patients with relapsed/refractory MM,^32^ CD19-CAR T cells were administered approximately 2 weeks after treatment with high-dose melphalan and autologous stem cell transplant (ASCT). The CAR construct included an anti-CD19 single-chain variable fragment linked to the 4-1BB and CD3ζ signalling domains.^7^ No severe CRS was observed, and most of the reported toxicity was attributable to the ASCT. Two patients showed significantly longer progression-free survival after CD19-targeted CAR T cell therapy was incorporated into the strategy, compared with prior high-dose melphalan and ASCT alone, prompting the authors to emphasise the possible additional use of CD19-CAR T cells in order to prolong the duration of response to standard myeloma treatment.^33,34^ Interestingly, CD19 expression on the myeloma cells was very low. Due to the inconsistent or absent expression of CD19 in the majority of patients with MM,^35^ the mechanism of action of CD19-targeted CAR T cells is controversial. Possible explanations for the positive results include the presence of a small population of CD19^+^ myeloma precursor cells, very low and undetectable CD19 expression on myeloma cells, and/or the eradication of non-malignant CD19^+^ B cells that might otherwise suppress the anti-tumour immune response.^36^

CD138 is a member of the syndecan family that is involved in cell–cell and cell–matrix interactions and is predominantly expressed on the surface of epithelial cells, plasma cells, and myeloma cells.^37^ Guo et al.^38^ designed a phase I clinical trial for relapsed/refractory MM patients using CAR T cells that target CD138. Results obtained from the five patients enrolled on this trial seem to be promising: four of the patients achieved stable disease for at least 3 months (range 3–7 months), whereas the fifth patient, whose MM had progressed to plasma cell leukaemia, showed a reduction in the level of myeloma cells in the peripheral blood. No relevant toxicities, particularly no epithelial damage, were observed.

Ramos et al.^39^ have constructed CAR T cells specific for the κ-light chain in order to target malignant cells in which κ-light-chain expression is restricted (e.g. in B cell and plasma cell malignancies) and to spare normal B cells expressing the non-targeted λ-light chain. In a phase I clinical trial including five patients with MM, the investigators reported that four of the patients achieved stable disease lasting 2–17 months.^39^

Natural killer group 2, member D (NKG2D) is an activating receptor usually expressed on the surface of immune cells, particularly on natural killer (NK) cells, T cells (γδ, and CD8^+^ and CD4^+^ subsets) and invariant NKT cells. NKG2D promotes the elimination of NKG2D-ligand expressing cells. On NK cells NKG2D serves as an activating receptor that triggers cytotoxicity upon ligand binding. In CD8^+^ T cells NKG2D promotes co-stimulatory signals.^40^ While absent in healthy tissues, the expression of NKG2D ligands (MIC-A, MIC-B and various UL16-binding proteins/RAET1 proteins) is upregulated in infected cells and various cancer types.^41^ T cells expressing an NKG2D-CAR construct induced significant anti-tumour activity in murine tumour models.^42^ Nikiforow et al.^43^ have demonstrated the safety and feasibility of a single infusion of NKG2D-targeted CAR T cells in five patients with relapsed/refractory MM in the first three dose-escalation cohorts in a phase I dose-escalation clinical study.

### Additional antigens investigated in preclinical CAR T cell studies

Further target antigens for MM CAR T cell therapies have been explored over the past few years, with recent studies particularly focussed on antigens such as signalling lymphocytic activation molecule F7 (SLAMF7; also known as CS1 and CD319) and CD38.

SLAMF7 is highly expressed in normal and neoplastic plasma cells and different immune cells (B, NK, NKT, T cells, dendritic cells and monocytes), but not in normal tissue parenchyma.^44–47^ Elotuzumab is a SLAMF7-directed mAb, which is currently under clinical investigation in newly diagnosed and relapsed or refractory MM.^48–50^ CAR-engineered cells specific for SLAMF7 are currently under development as both autologous and allogeneic ‘off-the-shelf’ approaches. Danhof et al.^51^ and Gogishvili et al.^52^ designed a SLAMF7-CAR construct derived from elotuzumab. SLAMF7-CAR T cells derived from MM patients and healthy donors demonstrated effective in vitro cytolysis of primary myeloma cells. Furthermore, in a xenograft mouse model SLAMF7-CAR T cells showed promising in vivo activity by significant reduction of medullary and extramedullary myeloma manifestations. Importantly, the SLAMF7-CAR T cells not only recognised MM cells but also selectively targeted SLAMF7^+^/-high B, NK, NKT and T cells, while sparing SLAMF7-negative/-low immune cells. The authors hypothesised that lymphocytic fratricide by SLAMF7-CAR T cells might lead to acute and chronic side effects such as cytokine storm and viral infections, when used in a clinical setting.^51,52^ Wang et al.^53^ also reported on a SLAMF7-CAR encoded by a lentiviral vector and containing a CD28 co-stimulatory domain. Two mutations on the IgG4 linker CH-2 portion were introduced to enhance the potency and persistence of the SLAMF7-CAR T cells. In vitro cell culture analysis revealed specific lysis of MM cell lines by SLAMF7-CAR T cells. In addition, compared to CARs directed against other antigens overexpressed by MM (BCMA and CD44v6 (CD44 adhesion receptor isoform variant 6), SLAMF7-CAR T cell administration resulted in the best anti-tumour activity in an MM-bearing mouse model.

These promising results support the suitability of SLAMF7-CARs for further evaluation in early-phase clinical trials.^53^ Furthermore, in terms of a potential universal ‘off-the-shelf’ approach, Galetto et al.^54^ and Mathur et al.^55^ designed allogeneic SLAMF7-CAR T cells derived from healthy donor peripheral blood mononuclear cells (PBMCs). As TCR-deficient T cells were demonstrated not to mediate alloreactivity in a xenograft-versus-host disease (GvHD) mouse model, the *TCRα constant* (*TRAC*) gene was inactivated to reduce the GvHD potential of the allogeneic SLAMF7-CAR T cells. Furthermore, the researchers aimed to minimise the risk of fratricide by *SLAMF7* gene inactivation in SLAMF7-CAR T cells. The allogeneic SLAMF7-CAR T cells specifically lysed MM cell lines and primary MM tumour cells. In an MM mouse model, a single injection of 10 × 10^6^ SLAMF7-CAR T cells resulted in a substantial decrease of serum monoclonal protein levels. Designing double-knockout (*TCRA* and *SLAMF7*) allogeneic SLAMF7-CAR T cells, the authors demonstrated the feasibility of the multiplex genome editing CAR approach, making the first steps towards the large-scale availability of SLAMF7-CAR T cells to MM patients.^55,54^

CD38 is frequently expressed on normal and aberrant plasma cells,^56–58^ and recent immunotherapeutic approaches have targeted CD38 with mAbs, including daratumumab, isatuximab, MOR03087, and Ab79.^59–63^ CD38 is not only found in plasma cells but also in lower levels on other immune cells such as B cells, B cell progenitors, NK cells, monocytes and hematopoietic cells.^64^ Drent et al.^65,66^ reported on CD38-CAR T cells based on three different CD38 antibodies, CD3ζ and 4-1BB signalling domains. Derived from healthy donor PBMCs, CD38-T cell CARs effectively lysed MM cell lines and primary MM cells. Significant in vivo anti-tumour effect was also observed in a xenograft mouse model. Furthermore, first steps towards safe clinical use were made by transduction of CD38-CAR T cells with a caspase-9-based inducible suicide gene that allowed effective control of CD38-CAR T cells. However, targeting MM cells with high-affinity CD38-CAR T cells also led to on-target, off-tumour effects, reflected by the lysis of normal CD38^+^ hematopoietic cells. To overcome this drawback, the authors used a ‘light-chain exchange method’, to produce CD38-CARs with up to 1000-fold lower affinity to CD38. Those low-affinity CD38-CAR T cells still effectively lysed CD38^+^ MM cells while little activity was observed against CD38^+  ^hematopoietic cells in vitro and in vivo. Through this, the authors successfully demonstrated that optimising CAR affinity is feasible, and may improve safety in clinical applications.^65,66^

CD44v6 was shown to be expressed in epithelial and haematologic tumours, including advanced high-risk MM.^67^ CD44v6 is also expressed on keratinocytes and immune cells as monocytes and T cells at low levels, and almost no expression is found on CD34^+^ haematopoietic stem cells.^68^ Casucci et al.^69,70^ aimed to target CD44v6 by a CAR construct containing a single-chain variable fragment (scFv) of humanised CD44v6 mAb, CD28 co-stimulatory domain and inducible caspase-9 suicide gene. CD44v6-CAR T cells showed long-term persistence and eradication of previously engrafted MM tumour cells in an in vivo mouse model, and no cytotoxic activity was observed against hematopoietic stem cells. The authors are currently designing a phase I/II trial of CD44v6-CAR T cell administration after ASCT.

CD229 has been demonstrated to be strongly expressed on MM cells and MM precursors, predisposing it as a potential therapeutic target.^71,72^ Radhakrishnan et al.^73^ generated the first humanised CD229 scFv, to create a CAR construct using a 4-1BB co-stimulatory domain. CD229-CAR T cells had strong cytotoxic activity against CD229^+^ MM cell lines and completely eradicated MM cells in a mouse model after only 18 days. Only limited toxicity was observed against other immune cells such as B and resting T cells. The authors plan to evaluate the efficacy of CD229-CAR T cells in relapsed/refractory MM patients in early-phase clinical trials.^73^

## NK cells for CAR-based immunotherapy in MM

Beyond the CAR T cell approach, recent studies have aimed to engineer NK cells using CAR technology, in order to increase their reactivity and recognition specificity towards myeloma cells. NK cells are effector lymphocytes of the innate immune system. NK cell cytotoxicity is regulated by signals from stimulatory (e.g. FcγRIII-mediated antibody-dependent cell-mediated cytotoxicity) and inhibitory receptors (e.g. killer-cell immunoglobulin-like receptors during downregulation of major histocompatibility class I (MHC I) on target cells). NK cells mediate cytotoxicity via perforin and granzyme release and the expression of apoptosis-inducing ligands (e.g., FasL and TRAIL).^74^ CAR NK cells could have several advantages over CAR T cells, including a presumed better safety profile, as well as a potentially higher anti-tumour activity due to their multiple activation modalities. Mature NK cells are short-lived cells, meaning no long-lasting toxicities are expected in NK cell-based CAR approaches, and suicide genes might not be required. Furthermore, although CAR expression would direct NK cells towards malignant cells, NK cell cytotoxicity can also be triggered in a CAR-independent manner via stimulatory and inhibitory receptors, presumably increasing the potential for anti-tumour activity.^74–76^

The generation of CAR NK cells directed against MM antigens and their in vitro (cell lines)/ex vivo (primary patient samples) anti-tumour activity has been demonstrated in several preclinical studies. Chu et al.^77^ successfully generated SLAMF7-targeted CAR NK cells that displayed MM cytolytic activity in vitro and ex vivo, as well as efficient suppression of tumour growth in an aggressive orthotopic MM xenograft mouse model.^77^ A CAR NK cell approach to target CD138 on MM cells was used by Jiang et al.^78^ who generated NK cells carrying a CAR consisting of an anti-CD138 scFv and CD3ζ. Furthermore, Leivas et al.^79^ produced NKG2D-targeted CAR NK cells by transducing autologous-activated and -expanded NK cells with NKG2D-CAR-containing 4-1BB and CD3ζ signalling domains. NKG2D-CAR NK cells showed significant cytotoxic activity against MM cells in vitro. These encouraging results warrant further development of CAR NK cells in preclinical and early-phase clinical trials for MM treatment.

## Genetically modified TCR approaches

**Fig. 1:**
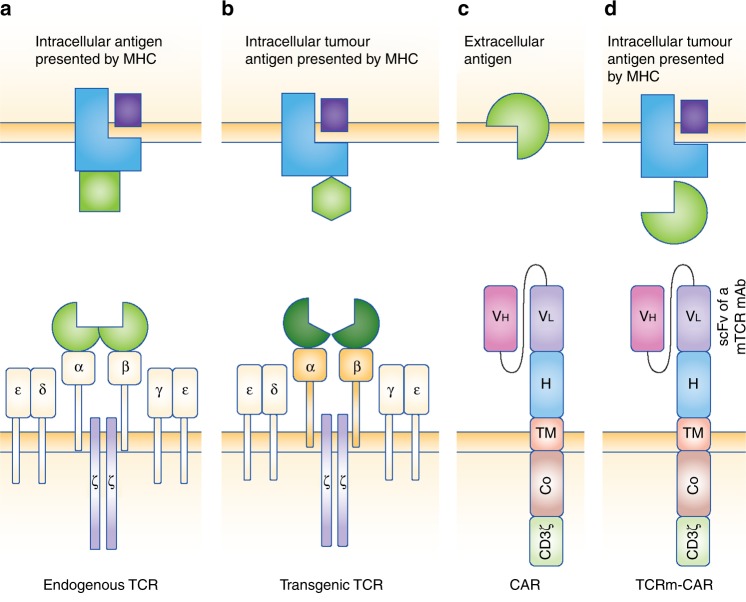
Principle structure of endogenous, engineered T cell receptor (TCR), chimeric antigen receptor (CAR) and TCR-mimic CAR. Endogenous and transgenic TCRs recognise intracellular peptides that are presented by the major histocompatibility complex (MHC). Additional co-stimulatory signals are required for complete T cell activation. **a** Endogenous TCRs consist of paired α and β chains (antigen recognition in context of MHC) associated with δ, ε, γ, and signalling ζ chains. **b** Transgenic TCR TCRαβ chains are genetically engineered to enhance or modify affinity. **c** Chimeric antigen receptors (CARs) recognise extracellular antigens independent of the MHC. The extracellular portion of the CAR consists of single-chain variable fragment (scFv) of a monoclonal antibody (heavy- and light-chain variable domains—V_H_/V_L_-specific for the targeted surface antigen) and a hinge region (H, stabilisation). The transmembrane domain (TM) serves as an anchor to the cell membrane. One or more intracellular co-stimulatory (Co, e.g. CD27, CD28, 4-1BB, OX40) and a CD3ζ chain domain represent signal transduction domains. **d** TCR-mimic antibody- (TCRm-) CARs are similar to the usual CAR constructs. Derived from monoclonal antibodies that mimic TCR function (TCRm mAb), TCRm-CARs thus recognise intracellular peptides presented on MHC I. Figure adopted from Fesnak et al.^81^

CARs provide a highly specific mechanism to target tumour antigens in an MHC-independent manner; however, they are limited to extracellular antigens. Addressing intracellular therapeutically relevant antigens might be advantageous in order to increase the number of tumour-derived targets, and cellular therapies based on engineered (transgenic) TCRs have been developed.^80^ Contrary to CARs, TCRs can recognise intracellular antigens presented by the MHC, and genetic modifications of TCRαβ sequences can be applied in order to change the affinity of the TCR, redirecting it towards therapeutically relevant antigens (Fig. 1). ^81^ Going one step further, TCR-mimic (TCRm) antibody CAR T cells were developed. These CAR constructs are similar to the usual CARs, but they are derived from antibodies that mimic TCR function by recognising peptides presented on MHC I (the so-called TCRm or TCR-like antibodies). TCRm-CAR T cells are therefore able to recognise intracellular antigens presented by human leucocyte antigen (HLA) haplotypes.^82,83^

Recent studies have demonstrated the feasibility and therapeutic efficacy of engineered TCR T cell approaches in malignant tumours.^84^ Furthermore, transgenic TCR T cell therapies are being investigated as an MM treatment, with current research focussing on targeting cancer testis antigens such as NY-ESO1, the B cell-specific transcription factor BOB1, or the intracellular transcription factor Wilms tumour-1 (WT1).

Several groups have developed engineered TCR and mTCR-CAR approaches to target NY-ESO-1, which was found to be highly expressed in poor-prognosis MM.^85–90^ Maruta et al.^85^ developed an mTCR-CAR T cell specific for an NY-ESO-1 that was presented by a HLA-A*02:01 molecule, and which showed significant anti-MM reactivity. Patel et al.^86,87^ demonstrated that NY-ESO-1 CAR T cell efficacy can be augmented by an NY-ESO-1^+^ T antigen-presenting cell (T-APC) vaccine. Superior anti-MM activity was observed in an MM mouse model when treated with both NY-ESO-1-CAR T cells and a T-APC vaccine, compared with CAR T cell treatment alone. Finally, clinical feasibility has been investigated by Rapoport et al.^89^ via a phase I/II clinical trial that focussed on targeting a peptide of NY-ESO-1 and of LAGE-1, which shows high homology to NY-ESO-1. Twenty antigen-positive MM patients with advanced disease were included and received autologous modified TCR T cells with enhanced affinity towards NY-ESO-1 and LAGE-1, following high-dose melphalan and ASCT. Upon a median follow-up of over 20 months, 14 (70%), 2 (10%) and 2 (10%) patients reached near/complete response, very good partial response and partial response, respectively. One patient (5%) had stable disease and 1 (5%) patient progressed. Overall, the authors demonstrated feasibility, safety, and promising anti-MM activity of NY-ESO-1-LAGE-1 TCR-engineered T cells.^89^

Aiming to target the intracellular B cell-specific transcription factor BOB1, Jan et al.^91–93^ isolated and transduced a TCR specifically recognising a peptide of BOB1 in the context of HLA-B*07:02 to recipient T cells. The transduced T cells efficiently lysed primary tumour cells of different haematological malignancies, including MM, and revealed potent anti-tumour response in an in vivo MM xenograft mouse model. As B cells also show BOB1 expression, significant B cell lysis was detected.^91–93^

Rafiq et al.^93^ designed a TCRm-CAR T cell that was directed against WT1, a transcription factor that was shown to be overexpressed in leukaemias and several solid tumours. In MM, WT1 was demonstrated to serve as an additional marker for risk stratification.^94^ The TCRm-CAR construct was derived from a monoclonal TCRm antibody (WT1 28z) recognising a specific peptide of the WT1 protein in the context of HLA-A*02:01. WT1 28z-CAR T cells showed WT1-HLA-A*02:01-specific cytotoxicity against various cancer cell lines, including MM. The authors aim to transfer this new adoptive mTCR-CAR T cell approach to the clinical setting for haematological and solid tumours.^95^

## Conclusions

Despite the advent of novel therapies and improved outcomes, patients with relapsed/refractory MM have a poor prognosis, and MM is usually an incurable disease. Improved treatment strategies are therefore needed for this malignancy. CAR T cell therapy appears to be a promising treatment option for MM. So far, the clinical use of CAR T cells is limited to a few antigens, early-phase trials, and patients with relapsed/refractory disease. Promising results with high response rates and manageable toxicities have been obtained in phase I/II trials using CAR T cells that target BCMA, CD19, CD138 or κ-light chain. Furthermore, preclinical studies on CD38-CAR T cells and SLAMF7-CAR T cells in the treatment of MM have yielded encouraging results that merit further investigation. Owing to these promising results, it is possible that CAR T cells will become a standard treatment option for relapsed/refractory MM over the next few years. Further investigation is needed in several areas, including identification of new targets, optimisation of CAR design to improve efficacy, and the combination of this treatment with other therapeutic approaches.
